# Notes and News

**Published:** 1894-07-14

**Authors:** 


### Notes and News.

Collected and Communicated.

Quite a new departure at Baltimore is projected; this is the organisation of coloured doctors, who propose to open a hospital and dispensary.

A number of new hospitals are projected in America for the treatment of railway employés. They are to be established at Cincinnati, Columbus, Cleveland, Indianapolis, and five other places. Employés with salaries exceeding £10 monthly will be taxed for their support.

Newport, in the United States, has had a recent important gift from Mrs. F. Vanderbilt. The sum of money is to be devoted to extending the hospital by adding a surgical building. It will contain a ward, operating theatre, and offices, and will be named the Vanderbilt Surgical Building.

A decided crusade is taking place against those morsels so adored by children in the street—namely, the halfpenny ice. Again is revealed another home of that busy, insatiable little enemy, the microbe. Both in Paris and London investigation proves that all manner of impurities find their way into these street ices.

Ladies who ride bicycles will welcome the champion who takes up their cause in a contemporary medical paper. The recent case of the woman who succumbed after a ride will naturally be used as an argument against the practice, feeble and unreasonable as such an argument is, as persons possessing weak hearts are liable to expire after no more exertion than a long walk entails. For the healthy, cycling is a health-giving mode of exercise, which must be admitted by those who have witnessed the marked improvement in those who have sought refuge from London atmosphere and noise, by mounting a bicycle and frequenting country lanes. It remains for the ladies to make their riding as graceful as possible, and to choose between health and pleasure on the one hand, and the general, though negative, approval which abstinence secures, from the majority of society.

At Oregon, in the United States, the Board of Medical Examiners have decided in the future to issue licenses to practice on stricter conditions, and two medical colleges in the State have resolved upon a compulsory four years' course of lectures. In the State of Oregon alone the Medical Register contains 746 names.

In an article on the state of medical education in Egypt, which appears in the *British Medical Journal*, Professor Virchow's criticism is quoted, and is of much interest. Speaking of the medical men, he says he has not met one who could be counted on to contribute in a useful manner to the progress of the science and art of medicine. The education itself, it would appear, is highly faulty. The teaching language is Arabic, and European teachers are not countenanced. The text-books are translations of works now out of date. Egyptian medical men possessing any pretentions to knowledge have received their education in France.

There is a patient at the present time at St. Mary's Hospital, whose misfortune should act as a warning to travellers on the Underground or, indeed, any railway. The warning should be taken to heart by those who, like the poor boy in question, stand too near an advancing train, and by those who, like the person who caused the accident, are in the habit of opening the doors of the carriages before the train has come to a standstill. This impatience on the part of a traveller to alight, caused him to open the door of the carriage which swept the boy standing within range, to the ground, when his arm was caught by the moving train and drawn between it and the platform. For nearly twenty minutes the poor child was fixed in this painful position whilst means were devised for his release, which was finally effected by sawing away a part of the platform. A broken arm and severe shock were much to pay for a slight act of carelessness, which is exceeded daily by thoughtless travellers, who only escape a like fate because they have not encountered the still more reprehensible person who swings open the door on entering a station regardless of those on the platform.

At the election of the Royal College of Surgeons the successful candidates were: Mr. Howard Marsh, of St. Bartholomew's Hospital, who polled 327 votes; Mr. Reginald Harrison, late of Liverpool Royal Infirmary, 266 votes; and Mr. James Hardie, of the Royal Infirmary, Manchester, 250 votes. About 500 Fellows voted. The annual meeting of Fellows was held after the election. The Council submitted a proposed alteration of bye-laws, and Mr. Frederick Treves moved, "That this meeting of Fellows of the Royal College of Surgeons of England cordially approves of increased power being given to the Council to deal with those Fellows or Members of the College who misconduct themselves, as contemplated in the proposed alteration of the bye-law, section XVI." The motion was carried unanimously. Mr. Treves, in moving the resolution, remarked that the noblest actions of the profession were often lightly passed over, but any act of misconduct on the part of a Fellow or Member was brought well forward and made much of, and the Council were accused of not doing its duty in not proceeding summarily against the offenders. But one had only to read the bye-laws to see that the Council had but very limited power, and in most cases were unable to interfere or remove the offending Fellow or Member from the roll of the College.

